# Ayurvedic Management of Presbycusis (Project TOPMAC): Protocol for an Exploratory Randomized Controlled Trial

**DOI:** 10.2196/55089

**Published:** 2024-09-23

**Authors:** Krishna Kumar V, Sanjeev V Thomas, Murugan C Nair, Parvathy G Nair, Nisha M L, Anuradha S, Arunabh Tripathi, Pallavi Mundada, Babita Yadav, B C S Rao, Sudhakar D, Srikanth N

**Affiliations:** 1 Central Council for Research in Ayurvedic Sciences New Delhi India; 2 Institute for Communicative and Cognitive Neurosciences Shoranur India

**Keywords:** presbycusis, Karnapurana, Kshirabala Taila, Rasayana, Withania somnifera (L.) Dunal, age-related hearing loss, hearing loss, sensorineural, auditory information, older adults, older adult, geriatrics, tinnitus, population-based, cognitive deterioration, topical oil pooling, efficacy, TOPMAC, intervention, hearing status, effectiveness, Ayurveda

## Abstract

**Background:**

Presbycusis is characterized by sensorineural hearing loss in both ears at high frequencies, which affects more than half of the older adults by the age of 75 years and is often accompanied by tinnitus and cognitive deterioration. Unfortunately, there are no treatments available to restore hearing loss. Treatment mainly focuses on improving the quality of life and communication with hearing aids. Traditional medicine like Ayurveda also explains ailments of a similar nature as Badhirya and advises using drugs with antiaging and neuroprotective activity for treatment. In Ayurveda, Badhirya and Karnanada (senile deafness with tinnitus) are due to vitiation of Vata Dosha. Treatments such as topical oil pooling (Karnapurana) are usually advised to counter Vata, improve hearing capacity, and reduce tinnitus. Kshirabala Taila, a medicated oil formulation prepared with *Sida cordifolia* Linnaeus, is one of the most preferred oils for topical oil pooling in such conditions, as it has a definitive indication for sensory dysfunctions. Drugs like *Withania somnifera* (L.) Dunal (Ashwagandha) are also used, as they ameliorate neurodegeneration and help to improve cognitive dysfunction.

**Objective:**

We propose an exploratory randomized controlled trial study for evaluating the efficacy of TOPMAC (Topical Oil Pooling with Kshirabala Taila and Supplementation of Ashwagandha Churna) in tinnitus suppression and hearing and cognitive function protection in patients aged 60-75 years with mild to moderate presbycusis.

**Methods:**

A parallel, 2-group, exploratory randomized controlled trial will be conducted in an Indian Ayurvedic research center at its outpatient service. Participants (N=60) with mild to moderate presbycusis will be recruited by screening. Participants will be randomized (computer-generated 1:1) to receive either basic treatment and health education (BTHE) or BTHE+TOPMAC for 24 weeks. The primary objective is to compare the efficacy of TOPMAC with that of BTHE in the protection of hearing function. The secondary objective is to compare the efficacy of TOPMAC with that of BTHE in tinnitus suppression and cognitive function protection.

**Results:**

This project was funded in January 2023. The institutional ethics committees at National Ayurveda Research Institute for Panchakarma (3/1/2020/NARIP/Tech/2036) and Institute for Communicative and Cognitive Neuro Sciences (IEC006) approved this study. The first patient was enrolled in September 2023; 22 participants were enrolled as of August 2024. The data analysis is yet to start, and the results are expected to be published by January 2025.

**Conclusions:**

If this exploratory trial is proven effective, it will steer the setting of a definitive randomized controlled trial to test whether the TOPMAC intervention can be incorporated as a cost-effective integrative approach for managing presbycusis. The Indian government has already launched a National Program for Prevention and Control of Deafness to benefit the deaf population. TOPMAC may later be considered for integration with the national program.

**Trial Registration:**

Clinical Trials Registry India CTRI/2023/04/051485; https://tinyurl.com/2h2hry3n

**International Registered Report Identifier (IRRID):**

DERR1-10.2196/55089

## Introduction

Presbycusis (age-related hearing loss) is characterized by sensorineural hearing loss at high frequencies in both ears, delayed central processing of auditory information, and problems understanding speech in loud environments [1]. It is usually associated with an audiogram that reveals the greatest hearing loss at higher frequencies. It affects over half of the older adults by the age of 75 years and nearly all older than 90 years [2]. Hearing loss is often accompanied by tinnitus. Many extensive population-based longitudinal studies have recommended that presbycusis is independently connected with cognitive deterioration.

The issues and effects of presbycusis are exacerbated in older adults due to additional degenerative processes in the central nervous system, which can lead to loss of neuroplasticity, cognitive ability, and other sensory modes, particularly vision [3]. Age-related hearing loss has an impact on both psychological well-being and physical ability. Some recent studies have established independent associations of hearing loss with driving ability, walking difficulty, social isolation, functional decline, and falls [4]. Social isolation causes emotions of incarceration and anxiety, which reduce higher cognitive performance, potentially increasing the economic and societal burden of age-related hearing loss [5]. The risk factors include age, male sex, diabetes mellitus, hypertension, and hereditary hearing loss [6]. Diabetes or hypertension, if not appropriately controlled, may also cause hearing problems through chronic arteriosclerosis. These are common chronic diseases in the aged population, leading to reduced blood flow in the inner ear [6]. Presbycusis is a serious but primarily neglected condition in India. The Indian government has started the National Program for Prevention and Control of Deafness to benefit the deaf population. The aims of this program include medically rehabilitating deaf persons of all age groups and fortifying the existing intersectoral linkages to maintain the rehabilitation program [7].

Unfortunately, there are no treatments available to restore lost hearing. Research on restoring hearing is a growing scientific field [8]. Treatment in most persons includes suitable hearing aids, but it is a limited solution, with some drawbacks. Studies have shown that people who would benefit from hearing aids are hesitant to use them due to cost, fit and comfort, maintenance, attitude, device factors, fiscal factors, psychosocial/situational factors, ear problems, and appearance. More precisely, these causes include the hearing aid’s ineffectiveness in loud environments, low benefit or low sound quality, and incompatibility with the type of hearing loss. Factors affecting hearing aid fit and comfort include the need for assistance in inserting and removing the hearing aid, feeling uncomfortable, or experiencing adverse effects (eg, rashes, itching) [9]. The treatment of tinnitus is also challenging [10]. Tinnitus should be managed using neurophysiological methods such as tinnitus retraining therapy—a combination of cognitive directive counseling and sound therapy, hearing aids, and white-noise generators. However, this retraining therapy also fails in 30%-40% of the patients [11]. A recent systematic review recommended that incorporating conventional or complementary and alternative therapies for treating mild cognitive impairment will be beneficial [12]. All functions of the nervous system in the human body are represented through Vata in Ayurveda. A subtype of Vata, Prana Vata, is situated in the head and controls intellectual functions, cardiovascular functions, sense organs, psychological activities, respiration, and reflex activities such as sneezing, belching, and deglutition [13]. Since presbycusis is a sensory dysfunction with cognitive decline, it is evident that the Prana Vata is impaired here. Ayurveda mentions similar ailments as Badhirya and Karnanada (senile deafness with tinnitus) [14]. The diseases Badhirya and Karnanada occur in the ears primarily by Vata Dosha vitiation. Topical oil pooling (Karnapurana) is a commonly employed treatment in such conditions, as it does the Vatashamana, improves hearing capacity, and reduces tinnitus. No mechanistic studies show the exact mechanism of action of Karnapurana. However, although histologically, the tympanic membrane is impermeable, a recent study [15] has discovered the possibility of a biological mechanism of active transport through the tympanic membrane to the middle ear. This way, the topically pooled medicine might be absorbed to impart its action in the inner ear. Ayurveda recommends a class of drugs termed as Rasayana, which possess antiaging properties that can be beneficial in these conditions. They can exhibit neuroprotective effects in the inner ear and brain tissue. Kshirabala Taila, a medicated oil prepared with *Sida cordifolia* Linnaeus, is preferred as the drug of choice, as it has definitive indications for sensory dysfunctions [16]. An animal study has also demonstrated that the aqueous root extract of *S. cordifolia* Linn has a neuroprotective effect due to its antioxidative properties and validated the Rasayana claim of *S. cordifolia* Linn in neurodegenerative diseases [17]. *Withania somnifera* (L.) Dunal (Ashwagandha) is another medicine commonly used, as it can regenerate damaged nerve cells and thereby improve nerve function [18]. Recent reviews have shown Ashwagandha as a potent medicine for cognitive function protection [19]. A randomized controlled trial has shown that Ashwagandha effectively enhanced immediate and general memory in individuals with mild cognitive impairment and improved attention, executive function, and information processing speed [20]. The administration of the water extract from Ashwagandha (100 mg/kg/d) with drinking water for 8 months has shown to be safe and showed no toxicity in rats [21]. Ashwagandha may operate as a gamma-aminobutyric acid mimetic, cholinomimetic, and agonist for α-7 nicotinic receptors for cognitive function protection [22]. However, no clinical trials or documented data are available on the efficacy of these drugs for presbycusis treatment, despite being widely used in clinics. The only available data is a case report recommending an Ayurveda treatment protocol to effectively improve hearing mechanisms in presbycusis with no side effects [23].

Hence, we propose an exploratory randomized controlled trial for evaluating the efficacy of TOPMAC (Topical Oil Pooling with Kshirabala Taila and Supplementation of Ashwagandha Churna) in tinnitus suppression and hearing and cognitive function protection in individuals aged 60-75 years with mild to moderate presbycusis and comparing this intervention with basic treatment and health education (BTHE) for presbycusis. BTHE includes treatment for diabetes mellitus, hypertension, and dyslipidemia and counsel related to lifestyle modification and avoiding alcohol and cigarette use. The primary objective is to compare the efficacy of TOPMAC with that of BTHE in the protection of hearing function. The secondary objective is to compare the efficacy of TOPMAC with that of BTHE in tinnitus suppression and cognitive function protection.

## Methods

### Trial Design

This is a multicenter, parallel, 2-group, exploratory, randomized (1:1) controlled trial. The plan of enrollment, interventions, and assessments is given in Table 1.

**Table 1:** Schedule of enrollment, interventions, and assessments for the TOPMAC (Topical Oil Pooling with Kshirabala Taila and Supplementation of Ashwagandha Churna) study.

			Screening		Baseline		Intervention period											End of treatment
Timepoint					Day 1		Day 29		Day 57		Day 85		Day 113		Day 141		Day 169	
	Potential participant identification and screening	✓																
	Provision of participant information sheet	✓																
	Eligibility assessment	✓																
	Informed consent	✓																
**Interventions**																		
	Ashwagandha Churna days 1-168			✓		✓		✓		✓		✓		✓				
	Karnapurana: weeks 1,5,9,13,17,21			✓		✓		✓		✓		✓		✓				
	Basic treatment and health education			✓		✓		✓		✓		✓		✓				
**Assessments**																		
	Demographics			✓														
	Tympanometry	✓																
	Pure tone average	✓								✓						✓		
	Speech recognition threshold	✓								✓						✓		
	Speech discrimination score	✓								✓						✓		
	Laboratory investigations	✓								✓						✓		
	Brainstem Evoked Response Audiometry			✓						✓						✓		
	Tinnitus Functional Index			✓						✓						✓		
	Montreal Cognitive Assessment			✓						✓						✓		
	Drug compliance (%)					✓		✓		✓		✓		✓		✓		
	Concomitant medication					✓		✓		✓		✓		✓		✓		
	Rescue medication					✓		✓		✓		✓		✓		✓		
	Adverse reactions					✓		✓		✓		✓		✓		✓		

### Setting

Likely eligible participants would be identified at the outpatient department of the National Ayurveda Research Institute for Panchakarma (NARIP). They will be sent for screening at the Institute for Communicative and Cognitive Neuro Sciences (ICCONS). NARIP is an Ayurvedic research hospital, and ICCONS is a medical hospital in rural Kerala, India.

### Eligibility Criteria

The intended population is adults aged 60-75 years diagnosed with mild to moderate presbycusis. Pure tone audiometry (PTA) will be performed for octave frequencies between 0.25 and 8 kHz, and the pure tone average will be calculated for 500 Hz, 1 kHz, and 2 kHz. The patients with a pure tone average of 26-55 dB hearing loss will be included. The other eligibility criteria are shown in Textbox 1.

**Eligibility criteria.**

**Inclusion criteria**

- Patients aged 60-75 years diagnosed with mild to moderate presbycusis

**Exclusion criteria**

- Conductive hearing loss and abnormal middle ear
- Current hearing aid user (>3 months)
- Patients with features of dementia
- Uncontrolled hypertension (systolic blood pressure >140 mmHg and diastolic blood pressure >90 mmHg)
- Uncontrolled diabetes (hemoglobin A_1c_>8%).
- Patients with very high dyslipidemia (total cholesterol >240 mg/dL, low-density lipoprotein >190 mg/dL, high-density lipoprotein >60 mg/dL, triglycerides >500 mg/dL) National Cholesterol Education Program Adult Treatment Panel III classification
- Patients with aspartate amino transferase and alanine amino transferase >2 times the upper normal limit
- Patients with serum creatinine >the upper limits of the normal
- History of malignancy within 5 years
- History of cerebrovascular accident within 1 year
- History of use of ototoxic, psychiatric, and nervous system drugs
- History of hypersensitivity to the trial drug or any of its ingredients
- Continuing addiction to smoking and usage of tobacco in any form
- Individuals simultaneously or previously (within 30 days before the investigation starts) participating in a clinical investigation using experimental drugs or devices

### Participant Identification

#### Screening

Individuals attending the outpatient department of NARIP identified as likely eligible to participate in the study will be given a participant information sheet. A trial team member will check initial eligibility if interested in participating. If the person is likely eligible, the researcher will send them to ICCONS for further screening.

#### Eligibility Confirmation

Eligibility confirmation will be done at ICCONS by an experienced audiologist and neurologist. This assessment will include tympanometry, PTA, and Montreal Cognitive Assessment (MoCA). Only patients with a pure tone average of 26-55 dB hearing loss will be included.

#### Consent

Written informed consent would be obtained before starting trial-related procedures or data collection. A suitably qualified and experienced person delegated by the principal investigator will get the consent. We have pilot tested the readability and comprehension level of the consent materials. For participants with literacy challenges and visual impairment, the bystander of the participant will help in the informed consent process.

### Randomization, Blinding, and Allocation Concealment

After giving consent, participants would be randomly chosen to receive either BTHE for 24 weeks or BTHE+TOPMAC in a 1:1 ratio using the serially numbered opaque sealed envelope provided by the Central Council for Research in Ayurvedic Sciences (CCRAS) headquarters. It is impossible to blind the participants or the investigators due to the trial design. The principal investigator enrolled the participants and assigned the intervention. However, the investigator conducting the audiometric evaluation is blinded. The statistician analyzing the collected data will also not be blinded to allocation.

### Interventions

#### Comparator

In BTHE, participants will receive treatment for diabetes mellitus, hypertension, and dyslipidemia and counsel related to lifestyle modification and avoiding alcohol and cigarette use. Advice on optimizing individual acoustic environments is provided, including reducing background noise and engaging in face-to-face conversations to maximize exposure to nonverbal communication cues. Lip reading classes will also be offered. This ancillary care is provided within the scope of this trial.

#### TOPMAC Intervention

The topical oil pooling will be done on weeks 1, 5, 9, 13, 17, and 21. The preparatory procedure includes temporal region massage with processed gingelly oil (Murchita Tila Taila) followed by sudation therapy. The main therapeutic procedure is to instill 5 mL of Kshirabala Taila in each auditory canal for 15-20 minutes in the morning. The therapy procedure includes massaging the ear base, and after 15-20 minutes, the oil will be wiped with dry cotton. Ashwagandha Churna will be supplemented with a dose of 6 grams at bedtime with 10 mL of cow’s ghee for 24 weeks. Participants in both groups are covered under clinical trial insurance.

### Study Training

A trained Panchakarma therapist will perform the topical oil pooling per the institute’s standard operating procedure (SOP). Trained technicians from ICCONS would perform all the audiometry evaluations. The investigator’s brochure and SOPs developed for the project are communicated to all the investigators. Further intermittent meetings of the investigators are planned. The reorientation/interaction with the study staff is repeated periodically during the investigators’ meeting.

### Concurrent Health Care for All Participants

All participants would be instructed to access routine health care, including medicines and consultations, with other health professionals. The details of the cointerventions would be noted at the assessments on day 29, day 57, day 85, day 113, and day 141.

### Primary Outcomes

The primary outcomes include differences in the average 500 Hz, 1 kHz, and 2 kHz in PTA from baseline and after the 24th week of intervention. PTA is the gold standard for measuring sensitivity and identifying the presence and degree of hearing loss. PTA (isolated frequency) over a range of frequencies critical for everyday listening can determine the degree, configuration, and type of hearing loss in a manner detailed enough to assist the health care team in determining the etiology and prognosis for the hearing loss as well as the optimal treatment strategy [24]. Pure-tone evaluation is typically performed in a sound-treated booth to reduce the impact of external sounds, with the booth environment and electroacoustic equipment calibrated to American National Standards Institute standards to optimize intertest and intratest reliability. Primary outcomes also include Brainstem Evoked Response Audiometry (BERA) changes and differences in speech recognition threshold and speech discrimination score.

### Secondary Outcomes

The secondary outcomes consist of differences in the tinnitus functional index (TFI) from baseline and after the 24th week of intervention. The secondary outcome also includes the MoCA score difference. TFI is considered the gold standard for measuring tinnitus severity. It is a 25-item questionnaire that identifies the tinnitus severity of the patient by calculating the overall TFI score. The 8 subscales address 8 critical domains of negative tinnitus impact: I, intrusive (unpleasantness, intrusiveness, persistence); SC, sense of control (reduced sense of control); C, cognitive (cognitive interference); SL, sleep (sleep disturbance); A, auditory (auditory difficulties attributed to tinnitus); R, relaxation (interference with relaxation); Q, quality of life (quality of life reduced); and E, emotional (emotional distress). In this way, the TFI score and subscore will measure the improvement in subjective well-being and quality of life related to tinnitus.

### Adverse Events

Any adverse event or adverse drug reaction, if seen during the trial period, will be documented suitably, and timely management would be performed, and the same would be reported to the ethics committee and the sponsor(s) at the earliest. Considering the participant’s age and the character of the intervention, predictable adverse events include gastrointestinal side effects (nausea, vomiting, and diarrhea), dizziness, fatigue, ear pain, itching in the ears, and fullness in the ears. Safety reporting would begin from the point of randomization and finish when the participant has finished their 169th-day assessment.

### Sample Size Determination

A previously published report on the administration of topical oil pooling in patients with presbycusis showed that the average hearing level for the frequency of 500 Hz, 1 kHz, and 2 kHz after treatment was 53 dB (for air conduction defect of the right and left ears) [11]. We based our sample size calculation on the assumption that the average hearing level at these 3 frequencies will be 50 dB in the TOPMAC+BTHE group, while it will be 60 dB in the BTHE group. A difference of 10 dB after treatment between both groups has been considered clinically meaningful. With a standard deviation of 12 dB, 80% power, and a confidence interval of 95%, the calculated sample size for each arm is 23. The sample size becomes 28.75, rounded to 30 per arm, adding an attrition rate of 25%. Therefore, 60 participants must be enrolled in the trial (30 in each arm). The attrition rate was based on the clinical experience in treatment compliance. Moreover, in a previous study [25] on Ayurveda interventions among older participants, such attrition had occurred. An interim analysis will be performed after 25% of the participants complete the study duration, and then the reassessment of the attrition rate will be considered along with the original sample size.

### Data Collection

#### Baseline Assessment

Baseline assessments include demographic data and Ayurveda parameters. It also includes assessing BERA, TFI, and MoCA score (Table 1).

#### Treatment Logs

For BTHE and TOPMAC intervention sessions, the drug compliance reports will record the date, clinician details, day of intervention, mode of delivery, and drugs issued.

#### Follow-Up Assessment

All participants will attend on day 29, day 57, day 85, day 113, day 141, and day 169 for follow-up. Drug compliance (%), concomitant medication, and rescue medication will be assessed at all visits. In addition to that, PTA, BERA, laboratory investigations, TFI, and MoCA score will be repeated on day 85 and day 169 (Table 1).

### Early Discontinuation/Withdrawal of Participants

During the study, participants can withdraw at any time. Moreover, the investigator may discontinue a participant from the study if they judge it indispensable for any reason, that is, if the patient is not ready to continue or is noncompliant with the study procedure (at least 80% compliance is crucial to continue in the study); if the patient develops a life-threatening crisis or any other severe sickness due to other pathology, which requires emergency treatment or making it necessary to introduce new treatment during the study period; or if the patient experiences any adverse event or adverse reaction requiring hospitalization. The decision to withdraw a participant from the study will be made only by the investigator, who will provide a thorough justification and indicate further management if needed. The sponsor and the ethics committee will be informed within 2 working days.

### Data Management

Data would be collected using paper case record forms (CRFs) and electronic CRFs and maintained by the principal investigator. Regularly monitoring data entry and data collection processes will be executed during the project. The electronic CRF for data entry will also be used to check data accuracy and validity. The modeling imputation technique will impute any outlier or missing data. The parameters having follow-ups will be imputed through the time series modeling, and those with single-point observations will be imputed with the population’s average or median depending on the variable’s distribution. The data will be collected and entered in the CRFs identified by the enrollment IDs only. The consent document and the screening and baseline information containing the participants’ names or identities will be secured more cautiously. The electronic format will be locked by password, and access to this data set will be restricted to authorized persons only. The shared data file will be locked with a separate code that can be accessed after the code is disclosed on request. The statistician at the CCRAS headquarters will analyze the data. After completion of the study, the data will be stored for at least 5 years.

### Statistical Methods

Data will be managed through SPSS software (version 29.0; IBM Corp). The data would be individually analyzed for central tendencies (mean, median), range, standard error, standard deviation, and 95% CIs for each intervention arm in each of the groups in the study. Data will be tabulated and graphically shown using a standard format, Microsoft Excel, and other software programs. For nonparametric values, the Wilcoxon signed-rank test and Mann-Whitney *U* test will be performed, and for parametric values, a paired 2-sided *t* test and Student *t* test will be done. Further, multivariable regression and Cox regression analyses will be used if any confounding factor or censored data are found during the analysis. Bayesian analysis may also be performed if any necessity arises after the initial phase of data analysis. This study will assess patients with mild to moderate presbycusis based on impairment. Clinically, it is established that participants with moderate impairment have a lesser chance of cure and persons with mild impairment have a higher chance of cure, which is the available information before the study starts. Therefore, we use this information as prior distribution to calculate the later treatment response of the participants. Further, Bayesian analysis gives another angle to explore the outcome and reduce the researcher’s dependency on *P* values.

Regression analysis will control the diversity during analysis and stratify results by demographic factors such as sex, socioeconomic status, and comorbidities. The analysis will be done by stratifying these factors to estimate the effect of the intervention as accurately as possible. The regression analysis will consider these factors to determine the intervention’s adjusted effect. In the case of dichotomous or multinomial type of data, a separate analysis will be performed for each stratum, or logistic regression analysis will be used.

### Data Monitoring

This study will be monitored by the Data and Safety Monitoring Board of the TOPMAC project at NARIP, Cheruthuruthy. A particular quality control program would be initiated to secure an agreement with the present approved protocol, good clinical practice, related regulations, and SOPs of NARIP. The principal investigator will develop data management and monitoring plans and manage the trial’s daily activities. The Data and Safety Monitoring Board team will perform quality assurance checks to ensure the integrity of randomization, study entry procedures, and data collection. The Data and Safety Monitoring Board team will conduct at least one e-Clinical Research Form inspection once a year. Additionally, the sponsor may monitor or audit the study per the approved protocol, good clinical practice, relevant regulations, and SOPs.

### Ethics Approval and Dissemination

The institutional ethics committees at NARIP (3/1/2020/NARIP/Tech/2036 dated January 31, 2023) and ICCONS (IEC006 dated March 21, 2023) approved this study (protocol-1 dated February 3, 2023). This study was registered on April 11, 2023 (CTRI/2023/04/051485). This protocol is reported according to the SPIRIT (Standard Protocol Items: Recommendations for Interventional Trials) statement [26] (completed SPIRIT checklist: Multimedia Appendix 1). Results would be published in a peer-reviewed journal with authorship eligibility according to the criteria of the International Committee of Medical Journal Editors.

## Results

This project was funded in January 2023. The institutional ethics committees at NARIP (3/1/2020/NARIP/Tech/2036 dated January 31, 2023) and ICCONS (IEC006 dated March 21, 2023) approved this study. The first patient was enrolled in September 2023; 22 participants were enrolled as of August 2024. The data analysis is yet to start, and the results are expected to be published by January 2025.

## Discussion

This randomized exploratory trial is proposed to assess the efficacy of TOPMAC intervention in improving the hearing status of adults aged 60-75 years with mild to moderate presbycusis.

### Strength

The TOPMAC intervention was methodically developed, amalgamating existing best and anecdotal shreds of evidence, and is the first trial to evaluate the effectiveness of Ayurveda intervention in presbycusis as an add-on to BTHE. The quantitative outcome measures included in this trial will give valid data about the possibility of performing a definitive randomized controlled trial of the TOPMAC intervention.

### Limitations

This study has limitations, as only effectiveness outcomes are included and not feasibility outcomes. This trial design has no follow-up period. The lasting effects of the treatment on presbycusis and associated symptoms may need to be assessed. Nevertheless, a case study has been reported, wherein the treatment effect of Ayurvedic medicines in presbycusis was sustained for a follow-up period of 6 months when no treatment was administered [23]. This study will apply only to Asian countries where Ayurveda is practiced. Nonetheless, the applicability of this protocol is limited in other continents. However, they could also replicate this model if there is accessibility of trained Panchakarma therapists to perform the Karnapurana procedure and if they have regulatory permissions to use Ashwagandha.

Another limitation is the absence of subjective Ayurvedic diagnostics in the research design, which might influence the broader integration of Ayurvedic treatments into conventional medical practice. However, Badhirya and Karnanada are described in Ayurveda and can be correlated with various symptoms of presbycusis. Although the standardized or validated assessment criteria for severity are not available in Ayurveda terminology, the outcome measures/tools for the assessment of presbycusis are conceptually applicable to inferring the results for Badhirya. Further, this protocol evaluates the patient’s prakriti (psychosomatic constitution) by using the standardized Ayur Prakriti web portal developed by CCRAS [27]. Prakriti is considered an essential part of Ayurvedic diagnostics [28]. Although TFI is a robust tool for assessing tinnitus impact, the absence of a broader quality of life measure for other dimensions possibly affected by the treatment is yet another limitation of this protocol. These limitations will be considered in the future definitive randomized controlled trial.

### Conclusion

If this exploratory trial is proven effective, it will steer the setting of a definitive randomized controlled trial, which will incorporate the limitations as described above, to test whether the TOPMAC intervention can be incorporated as a cost-effective integrative approach for managing presbycusis.

## Appendix

Multimedia Appendix 1 SPIRIT (Standard Protocol Items: Recommendations for Interventional Trials) checklist.

## Abbreviations

BERA: Brainstem Evoked Response Audiometry
BTHE: basic treatment and health education
CCRAS: Central Council for Research in Ayurvedic Sciences
CRF: case record form
ICCONS: Institute for Communicative and Cognitive Neuro Sciences
MoCA: Montreal Cognitive Assessment
NARIP: National Ayurveda Research Institute for Panchakarma
PTA: pure tone audiometry
SOP: standard operating procedure
SPIRIT: Standard Protocol Items: Recommendations for Interventional Trials
TOPMAC: Topical Oil Pooling with Kshirabala Taila and Supplementation of Ashwagandha Churna
